# Night eating syndrome is associated with mental health issues among palestinian undergraduate students-cross sectional study

**DOI:** 10.1186/s40337-022-00727-2

**Published:** 2023-01-03

**Authors:** May Hamdan, Manal Badrasawi, Souzan Zidan, Ruba Thawabteh, Raya Mohtaseb, Khozama Abu Arqoub

**Affiliations:** 1 Department of Health professions, Program of Healthy and Therapeutic Nutrition/Faculty of Medicine, Palestine Polytechnic University, Hebron, Palestine; 2 Department of Nutrition and Food technology, Faculty of Agriculture and Veterinary Medicine, An-Najah National University, PO. Box 7, West Bank Tulkarm, Palestine; 3 Department of Nutrition and Food technology, Faculty of Agriculture, Hebron University, West Bank Hebron, Palestine

**Keywords:** Night eating syndrome, Undergraduates, Prevalence, Mental health

## Abstract

**Background:**

University students are exposed to several factors associated with Night Eating Syndrome NES, which is distinguished by nocturnal consumption and/or evening hyperphagia. The main purpose of the current study is to examine the state of NES, and to explore its relationship with selected factors (e.g. sociodemographic factors, lifestyle habits, body mass index “BMI”, and mental health) among a sample of undergraduates.

**Methods:**

A cross-section design was done among undergraduates recruited from three universities in the southern part of Palestine. Students completed a self-administrated questionnaire including demographic information, lifestyle habits, medical profile, and the Arabic version of Night Eating Questionnaire (NEQ). Mental health status was also assessed using the Arabic version of the 12-item General Health Questionnaire (GHQ-12). Cronbach alpha was used to check the reliability of the Arabic version of NEQ. Data were analyzed using univariate and multivariate approach.

**Results:**

A total of 475 participants were included in the study, 197 (47%) males, 253(54%) females. Mean age was 19.8 ± 1.4 years, ranged from 18 to 25 years old. It is found that 141 university students (29.7%) screened positive for NES. According to univariate analysis, NES was significantly related to gender (*p* = 0.023), major (*p* = 0.005), personal monthly income (*p* = 0.007), source of funding (*p* = 0.005), and mental health (*p* < 0.005). Besides, the results of binary logistic regression revealed that having mental health problems (Exp (B) = 4.18; 95% CI = 2.50–6.98; *p* = 0.000), males (Exp (B) = 1.99; 95% CI = 1.17–3.39; *p* = 0.014), and those who study expenses was not covered either by scholarship or parents (Exp (B) = 2.75; 95% CI = 1.29–5.8; *p* = 0.08) were significantly associated with NES.

**Conclusion:**

It is found that NES is common among Palestinian university students. In this study, NES was significantly more prevalent among males, and those who were studying scientific majors, having a personal income between 500 and 1000 new Israeli shekel per month, and having mental problems, and those whose studies were funded by neither by a scholarship nor by their parents.

## Plain English Summary

Night eating syndrome (NES) is an eating disorder characterized by a time-delayed pattern of eating relative to sleep, where most food is consumed in the evening and night. There is evidence that adolescents had higher prevalence of NES in comparison to other age groups. Moreover, Former studies indicated that anxiety, stress, and depression may be correlated with NES and weight gain. The current outcomes showed that 29.3% of university students complying with symptoms consistent with the diagnostic criteria for NES. The findings also pointed out that gender, field of study, monthly income, source of funding, and mental health is a significantly associated with NES.

## Background

Night eating syndrome (NES), an eating disorder, is defined as a tardiness circadian pattern of food intake that was observed for the first time among obese people who were refractory to standard weight loss regimen [1]. It is featured by sleeplessness or insomnia (≥ 3 times/week), morning anorexia (negligible “i.e. juice or coffee” or no consumption at regular breakfast time), evening hyperphagia (eating a minimum of 25% of daily food intake post an evening meal), and snack consumption during night awakening (> 2weeks) [2].

Despite NES was not officially included in former versions of the DSM, for the first time, the suggested diagnostic criteria for NES were described in the Diagnostic and Statistical Manual of Mental Disorders 5th edition (DSM-5), and found its place under the section of “Other Specified Feeding or Eating disorders”[3]. However, there is no sufficient information about the etiology of NES [4].

The literature reports a wide range of NES prevalence, Among American university students the prevalence of NES was about 2.9% in 10 U.S universities [5]. He et al. (2017) noticed that the proportion of NES was 2.4% among Chinese college students [6]. Further study by Nolan and Geliebter done among 246 American undergraduates revealed that 5.7% of their study sample had NES [7]. Night eating syndrome was included in a systematic review of the prevalence of eating disorders [8], substantial variation in the prevalence of NES was documented, attributed mostly to the sample size, countries, instruments employed, the cutoff values, and differences in the studies design [8].

There are several factors associated with NES such as; depression, obesity, medications, gender, slowing down the habitual nighttime elevation in the leptin and melatonin levels, and high serum cortisol levels [5, 9, 10]. Furthermore, relationships between NES, maladaptive coping, poor psychosocial and physical functioning, and eating disorder behaviors and attitudes were reported [11, 12].

Disordered eating behaviors are more likely to occur in late adolescence [13], with a zenith between the ages of 18–20 years [14]. In particular, university students tend to have body image concerns [15], sleep disturbances [16], and anxiety and stress [17], all of which increases the risk of developing NES symptoms. Striegel-Moore and his colleagues reported that the presence of evening hyperphagia appears to be more common in young adults (18–30 years old) in comparison to the general population [18].

So far, the majority of former studies investigating NES were done in Western nations, moreover, there is no published studies that have estimated the prevalence of NES among Palestinian university students. In a view of the fact that cultural and social background is an fundamental contributing factor associated with occurrence of eating disorders (e.g. Anorexia nervosa, and binge eating) [19, 20], so the prevalence and related factors of NES could be probably distinct in Palestine from those reported in the Western nations.

Accordingly, the main purposes of the current study is to estimate the proportion of students who comply with behaviors and symptoms consistent with the diagnostic criteria for NES in a sample of undergraduate students in three Palestinian universities, and to examine whether or not certain variables (e.g. sociodemographic characteristics, lifestyle habits, medical profile, and mental status) are associated with the development of NES.

## Methodology

### Study design, settings, and population

The current study used a cross-sectional design. The study population was university undergraduates students from the southern part of West Bank, Palestine. The three Palestinian universities in the southern West Bank were formally approached through formal processes. After obtaining authorization, the research team visited university campuses during working days, and students who were accessible on campus were invited to participate in the study. During the data collection period, the total number of students from the three universities was 17,000 students.

### Sample size calculation and sampling method

Random sampling method was used to recruit university students. The sample size was determined using a single proportion for a finite population. Sample size calculations was done using G power software with an alpha of 0.05 (two-sided) and 80% power, Prevalence of night eating syndrome from previous research used the NEQ as an instrument to NES in a similar study population [4], indicated that a minimum of 200 participants was needed to determine a prevalence of NES. To determine the association between NES and mental health and other sociodemographic factors, the sample size was recalculated considering the Chi square and regression are the required statistical test, 5% level of significance, (80%) power, giving a sample size of 450 participants. Considering drop out the sample size was increased to 500 participants.

The inclusion criteria were university students aged between 18 and 22 years old, and willing to participate and to provide all the required data. While the exclusion criteria involved pregnant and breastfeeding women, having mental illness that may limit their ability to fully answer the questionnaire, rejecting to participate in the study, refusing to sign a written consent, and having incomplete responses.

### Ethical considerations

The research protocols were in accordance with the Declaration of Helsinki and reported in line with the STROBE checklist for reporting cross-sectional studies. The study protocol was approved by the Deanship of Scientific Research Ethical Committee at Palestine Polytechnic University (reference number KA/41/2021). Informed written and verbal consent was collected from all university students prior to data collection.

### Data collection and research tool

Data collection was done face-to-face by using a pre-tested questionnaire, which was divided into five sections; (1) demographic characteristics, (2) medical profile, (3) lifestyle habits, (4) mental health, and (5) night eating syndrome. The team of four researchers collected the data within four months starting from December 2021 till March 2022. University students were verbally briefed about the purpose of study, and they were also informed about the type of data that would be collected, with affirmation on the optional participation. University students who agreed to sign the written consent were included in the data collection.

#### Demographic characteristics

The collected sociodemographic characteristics were university name, faculty name, academic year, gender, age, living place, marital status, personal income, and source of funding. University students were also asked to self-report their weights and height to assess university students’ nutritional status. Body mass index was calculated as (body in kilogram divided by height squared in meter (kg/m^2^), thereafter classified according to WHO cut off points [21].

#### Medical profile

In this section, health status was determined by asking university students about suffering from chronic diseases; if yes (name the disease, and the duration from suffering from it), undergoing a previous surgery; if yes (when?), and using medicines on regular basis; if yes (name the medicine and the purpose of its use).

#### Lifestyle habits

Lifestyle habits data included questions about smoking; are you smoker?; if yes (how long did you smoke? are you a shisha smoker or cigarettes smoker? How many times do you smoke cigarettes or shisha per week/day? University students’ physical activity level was also assessed using the short version of international physical activity questionnaire (SF-IPAQ) [22].

#### Mental health status

University students’ mental health status was measured using the 12-item general health questionnaire (GHQ-12) [23]. The validity and reliability of the Arabic version have been confirmed[24]. GHQ-12 scores were calculated using the GHQ scoring system, where: 0 = better than usual, 1 = same as usual, 2 = less than usual, and 3 = much less than usual. The cumulative score ranges from 0 to 36, with higher scores indicating higher degrees of disturbance of the general health status. University students scoring 15 points or higher were considered to have a tendency toward psychological problems [25, 26]. The GHQ questionnaire had good reliability in this study, with a Cronbach alpha of 0.81.

### Night eating questionnaire (NEQ)

A formerly validated and published NES questionnaire (NEQ) was adopted to assess NES in university students. NEQ is of 14-items questionnaire with a 5-point Likert scale [27]. According to the knowledge of the researchers, there was no verified Arabic version of the NES tool. As the NEQ is the most popular and reliable instrument for NES screening, it is widely employed. Following approaches were used to modify the questionnaire. Before translating the NEQ, we examine the content validity to determine if the NEQ is compatible with The Palestinian culture. The content validity was determined with the aid of five specialists; three nutrition specialists, one clinical psychology specialist, and one human sciences (assessment) specialist opted to use the instruments since they are compatible with Palestinian culture and behavior. The questionnaire was translated into Arabic utilizing the repeating “forward-backward” technique by an authorized translator. A pilot research with 30 participants was also conducted to assess if there were any difficulties in completing the questions; however, the Cronbach’s alpha was only performed on the whole data set. NEQ questionnaire obtained a good reliability, with a Cronbach alpha of 0.71. NEQ employing a clinical cut-off score of 25 for a broad assessment and 30 for a higher level of specificity [4]. Both values were used in the literature, and this variance has an effect on the prevalence of NES. As we are doing a screening study, we utilized a clinical cut-off score as 25.

### Data analysis

The data analysis was done using the statistical package for the social sciences SPSS version 21. Continuous variables were assessed for normality of distribution graphically and via the Shapiro-Wilk Test. Descriptive analysis including the means and standard deviation were used to analyze continuous variables, while categorical variables were described in percentages and frequencies. Mann- Whitney test was used to investigate the relationship between continuous variables and NES. On the other hand, Chi-Square test was used for analyzing the association between NES and categorical data. Significant level was set at p < 0.05. Further analysis was done using binary logistic regression to determine the risk factors for NES in a multivariate model, all the variables that showed significant association with NES in the univariate analysis p < 0.05, were included in the model. We checked logistic assumptions, multicollinearity, and outliers. To determine how well the model fit the data, we used the Hosmer–Lemeshow goodness of-fit test.

## Results

### University students’ recruitment

**Fig. 1:**
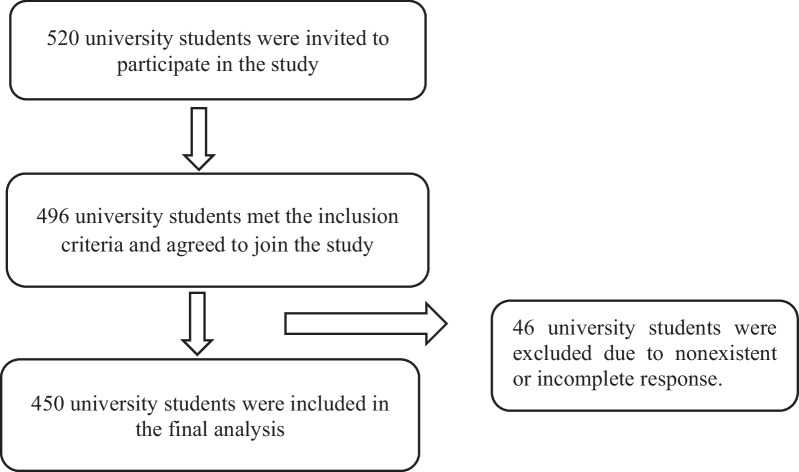
University students’ recruitment steps

University students were recruited from three Palestinian universities including; Hebron University, Palestine polytechnic University, and Bethlehem University. A total of 520 university students were invited to participate in the study, of whom 496 participants met the inclusion criteria and signed a written consent to join the study. Only 450 participants were included in the final analysis: 253 (56.2%) females and 197 (43.8%) males. The rest of the participants were excluded mainly due to nonexistent or incomplete responses as shown in Fig. 1.

### University students’ sociodemographic characteristics

A total of 450 university students aged 18–25 years old (*M* = 19.8, *SD* = 1.4) were analyzed (Table 1). 185 university students (41.1%) were studying at Hebron University in Hebron city, 64.2% of them were studying sciences majors, and 32.9% of them reported that they were in the first academic year. Our analysis reveals that a little more than the half were living in the city (59.6%), and the majority were living their families (96.0%), and were unmarried (94.7%). The personal income for around 68.0% of them was between 500 and 1000 Israeli shekel per month. The data also depicts that the parents was the source of study funding for the vast majority of the university students (89.8%).

**Table 1:** University students’ sociodemographic characteristics according to their gender presented in numbers (n) and percentages (%)

Variables		Males (*n* = 197)		Females (*n* = 253)		Total (*n* = 450)	
		n	%	n	%	N	%
University	Palestine Polytechnic University	87	44.2	92	36.4	179	39.8
	Hebron university	84	42.6	101	39.9	185	41.1
	Bethlehem university	26	13.2	60	23.7	86	19.1
Major	Social Sciences/ Humanity	56	28.4	67	26.5	123	27.3
	Sciences	119	60.4	170	67.2	289	64.2
	Applied Professions	22	11.2	16	6.3	38	8.4
Academic year	1st year	59	29.9	89	35.2	148	32.9
	2nd year	56	28.4	46	18.2	102	22.7
	3rd year	41	20.8	68	26.9	109	24.2
	4th year	36	18.3	48	19.0	84	18.7
	5th year	5	2.5	2	0.8	7	1.6
Place of residence	City	103	52.3	165	65.2	268	59.6
	Village	79	40.1	77	30.4	156	34.7
	Camp	15	7.6	11	4.3	26	5.8
Living status	With the family	188	95.4	244	96.4	432	96.0
	University accommodation	9	4.6	9	3.6	18	4.0
Marital status	Unmarried	195	99.0	231	91.3	426	94.7
	Married	2	1.0	22	8.7	24	5.3
Personal monthly income (Israeli shekel)^a^	< 500	114	57.9	192	75.9	306	68.0
	500–1000	32	16.2	30	11.9	62	13.8
	> 1000	51	25.9	31	12.3	82	18.2
Source of funding	Scholarship	4	2.0	7	2.8	11	2.4
	Parents	163	82.7	241	95.3	404	89.8
	Others	30	15.2	5	2.0	35	7.8

^a^US $ 1 ≈ 3.4 Israeli shekel

### University students’ lifestyle habits, medical history, and nutritional status

**Fig. 2:**
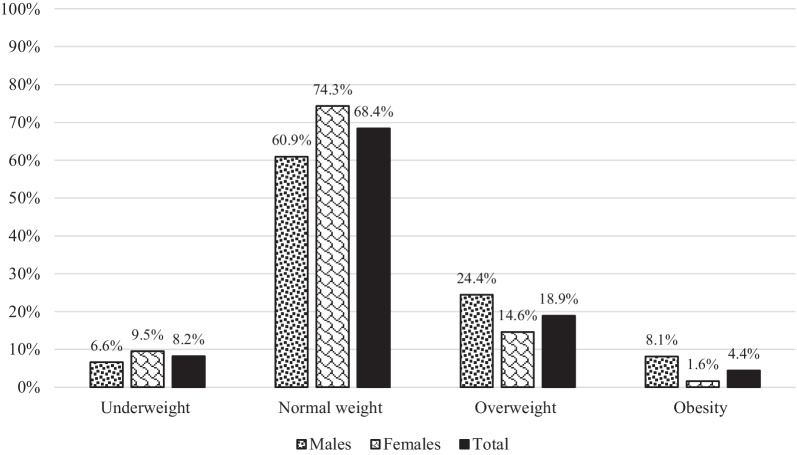
Weight categories of university students based on body mass index according to gender

Lifestyle habits data indicates that 67 university students (14.9%) were regular smokers with a mean duration of 4.0 ± 3.2 years, ranged from 1 to 24 years. Furthermore, the analysis shows that university students smoke cigarettes by a percentage of 19.6%, and smoke nargilah by a percentage of 18.9%. According to IPAQ, university students were classified as sedentary individuals by 43.0%, moderately active individuals by 31.8%, and very active individuals by 25.1%. In terms of university students’ medical history, the vast majority of them stated that they hadn’t chronic diseases (96.2%), didn’t take medicines on regular basis (92.4%), and hadn’t underwent a previous surgery (85.8%). Figure 2 shows that 68.4% of university students had normal weight, 18.9% were overweight, whilst 4.4% of the current sample were obese.

### University students’ mental health

**Fig. 3:**
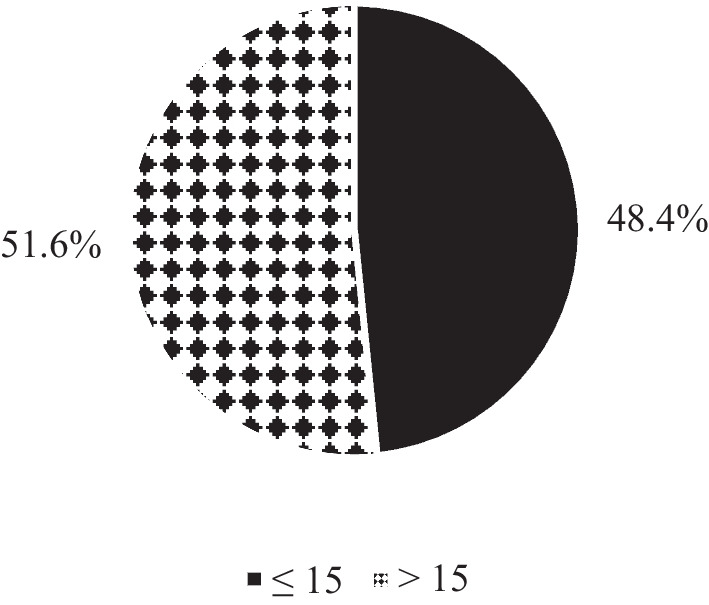
University students’ mental health presented in percentages

The mental health of university students is presented in Fig. 3. The mean of the GHQ score was 16.4 ± 6.4 ranging from 1 to 35 points. For the GHQ subscales; the mean of psychological distress (e.g. depression, anxiety) was 5.9 ± 3.4 ranging from 0 to 15 points, for social and emotional dysfunction the mean was 7.2 ± 2.9 ranged from 0 to 15 points, the mean for cognitive disorder was 3.4 ± 1.6 ranged from 0 to 6 points. According to Figs. 3 and 232 university students (51.6%) had mental problems.

### Prevalence of NES and its associated risk factors

**Fig. 4:**
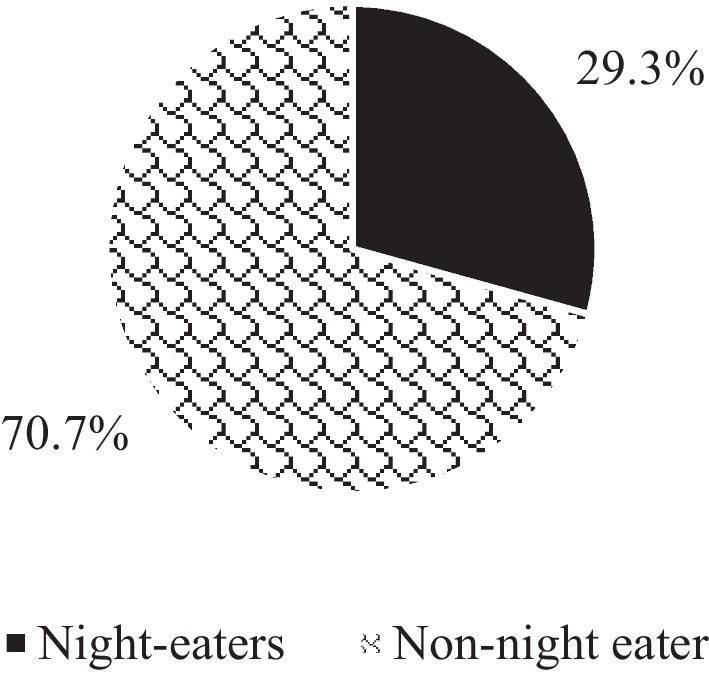
Prevalence of NES

The mean of the NES score was 20.2 ± 7.2 ranging from 6 to 39 points, and one-fourth of university students (29.3%) reached the criteria for NES as shown in Fig. 4. According to univariate analysis, there were no significant differences between night eaters and non-night eaters groups in academic year, age, place of residence, living status, marital status, BMI, medical history, or lifestyle habits (Table 2). The results also revealed that a significantly higher prevalence of NES was reported in university students studying at the faculty of medicine and health sciences, being female, having a personal monthly income ranging from 500 to 1000 NIS per month (*p* < 0.05). furthermore, source of funding was significantly correlated with NES (*p* < 0.05), indicating that university students whose studies were funded by source other than their parents or scholarship were more likely to have NES compared to the others. Moreover, the data analysis reveals that university students with NES were significantly more likely to have mental problems compared to those without NES (*p* < 0.05).

Table 3 presents the findings of the binary logistic regression of factors associated with NES in the current sample. The factors associated with NES were: having mental health problems (Exp (B) = 4.18; 95% CI = 2.50–6.98; *p* = 0.000), males (Exp (B) = 1.99; 95% CI = 1.17–3.39; *p* = 0.014), and those who study expenses was not covered either by scholarship or parents (Exp (B) = 2.75; 95% CI = 1.29–5.8; *p* = 0.08).

**Table 2:** The occurrence of NES by university students’ characteristics

Variable		Night eaters (*n* = 141)	Non- night eaters (*n* = 334)	*p*-value
University	Palestine Polytechnic University [*n* (%)]	42 (23.5)	137 (76.5)	0.077
	Hebron university [*n* (%)]	63 (34.1)	122 (65.9)	
	Bethlehem university [*n* (%)]	27 (31.4)	59 (68.6)	
Major	Social sciences/ Humanity [*n* (%)]	49 (39.8)	74 (60.2)	0.011
	Sciences [*n* (%)]	73 (25.3)	216 (74.7)	
	Applied professions [*n* (%)]	10 (26.3)	28 (73.7)	
Academic year	1st year [*n* (%)]	42 (28.4)	106 (71.6)	0.821
	2nd year [*n* (%)]	33 (32.4)	69 (67.6)	
	3rd year [*n* (%)]	28 (25.7)	81 (74.3)	
	4th year [*n* (%)]	27 (32.1)	57 (67.9)	
	5th year [*n* (%)]	2 (28.6)	5 (71.4)	
Age [years; mean ± SD]		1.4 ± 0.13	1.4 ± 0.08	0.970
Gender	Male [*n* (%)]	69 (35.0)	128 (65.0)	0.013^*^
	Female [*n* (%)]	63 (24.9)	190 (75.1)	
Place of residence	City [*n* (%)]	73 (27.2)	195 (72.8)	0.389
	Village [*n* (%)]	52 (33.3)	104 (66.7)	
	Camp [*n* (%)]	7 (26.9)	19 (73.1)	
Living status	With the family [*n* (%)]	127 (29.4)	305 (70.6)	0.559
	University accommodation [*n* (%)]	5 (27.8)	13 (72.2)	
Marital status	Unmarried [*n* (%)]	125 (29.3)	301 (70.7)	0.594
	Married [*n* (%)]	7 (29.2)	17 (70.8)	
Personal monthly income (Israeli shekel)	500 and below [*n* (%)]	85 (27.8)	221 (72.2)	0.006^*^
	500–1000 [*n* (%)]	12 (19.4)	50 (80.6)	
	1000 and above [*n* (%)]	35 (42.7)	47 (57.3)	
Source of funding	Scholarship [*n* (%)]	3 (27.3)	8 (72.7)	0.003^*^
	Parents [*n* (%)]	110 (27.2)	294 (72.8)	
	Others [*n* (%)]	19 (54.3)	16 (45.7)	
BMI categories	Underweight [*n* (%)]	13 (35.1)	24 (64.9)	0.841
	Normal weight [*n* (%)]	90 (29.2)	218 (70.8)	
	Overweight [*n* (%)]	24 (28.2)	61 (71.8)	
	Obesity [*n* (%)]	5 (25.0)	15 (75.0)	
Smoking	Regular smoker [*n* (%)]	25 (37.3)	42 (62.7)	0.273
	Irregular smoker [*n* (%)]	18 (25.7)	52 (74.3)	
	Non-smoker [*n* (%)]	89 (28.5)	223 (71.5)	
Cigarette smoking	Yes [*n* (%)]	32 (36.4)	56 (63.6)	0.076
	No [*n* (%)]	99 (27.8)	257 (72.2)	
Nargilah smoking	Yes [*n* (%)]	25 (29.4)	60 (70.6)	0.549
	No [*n* (%)]	106 (29.5)	253 (70.5)	
Physical activity level	Sedentary [*n* (%)]	55 (28.6)	137 (71.4)	0.792
	Moderate [*n* (%)]	41 (28.9)	101 (71.1)	
	High [*n* (%)]	36 (32.1)	76 (67.9)	
Had chronic disease	Yes [*n* (%)]	3 (17.6)	14 (82.4)	0.216
	No [*n* (%)]	128 (29.7)	303 (70.3)	
Underwent surgery	Yes [*n* (%)]	19 (29.7)	45 (70.3)	0.526
	No [*n* (%)]	113 (29.3)	273 (70.7)	
Taking medicines regularly	Yes [*n* (%)]	10 (29.4)	24 (70.6)	0.565
	No [*n* (%)]	122 (29.3)	294 (70.7)	
Mental health	Normal mental health [*n* (%)]	91 (39.2)	141 (60.8)	0.000^*^
	Mental problems [*n* (%)]	41 (18.8)	177 (81.2)	
Psychological distress [mean ± SD]		7.0 ± 3.31	5.4 ± 3.30	0.000^*^
Social &emotional dysfunction [mean ± SD]		7.9 ± 3.12	6.9 ± 2.77	0.001^*^
Cognitive disorder [mean ± SD]		3.7 ± 1.50	3.2 ± 1.64	0.002^*^

Data are presented as *n* (%) or mean ± SD; * *p* < 0.05. Pearson chi-square test is employed for categorical variables and Mann–Whitney test for continuous variables. SD: standard deviation.

**Table 3:** Binary logistic regression of risk factors associated with NES

Factors	Exp (B), CI	*p*-value	*Exp B*
Mental health			2.81 *
Having mental health issue	4.18 (2.50–6.98)	0.000*	
Major			
Being in social sciences/ Humanity	2.25 (0.847–2.02)	0.103	
Gender			
Being male	1.99 (1.17–3.39)	0.014^*^	
Personal monthly income			
1000 and above	1.2 (0.91–1.76)	0.08	
Source of funding			
Not covered by scholarship or parents	2.75 (1.29–5.8)	0.008^*^	

^*^Significant at *p* < 0.05. CI Confidence interval

## Discussion

To the best of our knowledge, the current study was the first to estimate the prevalence of NES, and to explore the relationship of NES with sociodemographic characteristics, medical history, lifestyle habits, BMI, and Mental health among Palestinian university students. Because this study was observational, clinical interviews were not carried out to diagnose NES; rather the night eating questionnaire (NEQ) was used to assess the percentage of students who reported behaviors and symptoms consistent with the diagnostic criteria for NES.

In the analysed sample, many university students had a high NEQ scores. By using the NEQ ≥ 25 cut-off point, the prevalence of NES among the current sample reached 29.7% higher than the evaluated proportion of NES for the general population which was 1.1% [28], and higher than the evaluated prevalence for other countries of university students; 5.8% Egypt [29]Turkey 9.5% [4], United states 4.2% [5], China 1.6% [6] and Brazil 15% [30]. Even though the aforementioned research utilized the same instrument and cut-off values, the outcomes varied significantly. This disparity is mostly attributable to variances in each community’s way of life, the learning and teaching approaches at colleges, socioeconomic status as well as the sample size. It is important to note that the current study was conducted following the COVID lockdown, which had a significant impact on Palestinian university students’ eating habits and lifestyle [31]. This high prevalence of NES, according to the authors, may be related to the fact that students are still impacted by these changes. Moreover, This result may be attributable to the availability of late-night restaurants in Palestine, which permits students, to dine out at night to converse or watch sports games while ordering food.

Other possible explanations for the high values of NES include the fact that students with higher personal incomes recorded a significantly larger proportion of NES (42.7%). As well as students who do not have a scholarship or financial help from their parents, a larger proportion of NES (54.3%) was recorded. As it was noticed that NES was more likely to occur among students whose studies were funded by means other than parents or scholarships. Other means can be the student himself, and in this case the student has to work in the night in order to fund his studies. Hence, working in the night while studying during the day puts a lot of pressure or maybe anxiety on the student leading to nocturnal eating. This could explain the current result.

Both indicators may suggest that these students are working at night to earn money, which may influence their eating habits and cause them to become night eaters.

The vast majority of NES assessments among university students propose that the percentage of its occurrence is higher than in the general population. Except for one German study reported that the prevalence of NES among a sample of students was within the range of 1.1 to 1.5%, which is prospected for the general population [32]. As the literature lacks studies conducted inside the Palestinian community, a comparison between university students and Palestinians requires additional investigation. This finding may also explained by the availability of late hour restaurants in Palestine allows students to dine out conveniently at night to chat together or watch sports games together in a group while ordering food to eat, especially among males.

Regarding the relationships between university students with NES and those with no NES, the current findings revealed that there were no significant differences in terms of BMI, physical activity, and smoking status. Similar results were also indicated in former studies [5, 18, 29, 33].

There are established theories explain the relationship between night eating practices and weight gain[34]. A comparison of the circadian rhythms of leptin, ghrelin, glucose, and insulin in people with NES and healthy controls revealed disruptions in the rhythms of leptin, ghrelin, glucose, and insulin among night eaters as compared to normal [35]. This disruption has been linked to obesity, and metabolic irregularities cause changes in physiological systems that might have a negative impact on health.[34] Despite these findings, conflicting data linking NEs and BMI has been presented. Bruzas and Allison (2019) reviewed 11 studies that investigated the relationship between NES and BMI, five of these studies indicated a significant association between the two variables, five demonstrated that there was no relationship, and one provided mixed data. [36] A Negative eating habits indicate long-term weight gain and a high BMI. [37] As the current study sample consists of young individuals, it appears that their eating behavior has not yet affected their weight. Other possible explanations for the lack of association include energy balance, overall caloric intake, and the caloric density of foods consumed at night.

Another notable finding in this study is that there is a statistically significant relationship by using univariate and multivariate analysis between gender and NES prevalence among the studied students as it was noticed that males had a relative higher occurrence of NES than females. This result comes in agreement with former studies done by He et al., (2017) [6], and Colles et al., (2007) [38], however, it is inconsistent with other studies that indicated no gender differences [4, 7, 32]. Since gender composition was not evenly distributed in the current sample, the result of a significant association between NES prevalence and gender may necessitate further studies in the future.

Besides, students from scientific background possessed a higher risk of developing NES than students with humanities background was found. The finding contraindicates a Malaysian study which found that students who were studying technical majors were at a greater risk of developing NES compared to students who were studying either scientific or humanities majors.

Both multivariate and univariate analysis showed that NES occurrence were found to be significantly associated with mental problems (psychological distress, emotional dysfunction, and cognitive disorder). This is in line with the findings from former studies [4, 6, 30]. It was found in a former study conducted on 849 Chinese college students that the prevalence of NES was significantly and positively related to psychological distress [6]. Furthermore, Sevincer and his colleagues that NES scores was significantly and positively associated with depression and anxiety symptoms [4]. In addition, the emotional state of Brazilian university students was significantly associated with the behaviors of NES syndrome [30]. Our finding indicates that it is relatively prevalent for students with NES to have mental problems (psychological distress, emotional dysfunction, cognitive disorder). However, it stills obscure whether mental problems is a clinical feature, cause, or a consequence of NES. Further studies are warranted to explore the exact relationship between mental problems and NES.

The study has several limitations that should be mentioned. First; since the current study is considered a cross-sectional, it does not permit casual inferences. Second, since the current study only included undergraduates from three universities in the southern part of Palestine, therefore the findings might not be representative for undergraduates in the whole Palestine. Third, the collected data was self-reported, thus there may be incorrect or biased data provided by undergraduates. Lastly, undergraduates’ participation were self-chosen and not compulsory. This sampling technique may result in a “selection bias” concerning undergraduates’ attention in completing the questionnaire as undergraduates with disordered eating pattern may not have participated in this study, giving rise to underestimation in the proportion of undergraduates having symptoms congruent with NES in the current study. Nonetheless, this study is the first of its kind in providing a worthy data about the prevalence of NES among university students and its’ association with lifestyle variables. Future studies should focus on clarifying the causal relationship, and assess nutritional for a better understanding of the NES. Further studies should also be conducted on a large sample size.

## Conclusion

To conclude, the current study reported a high prevalence of NES among Palestinian university students. It was also found that university students were at risk of NES especially being a male, studying in scientific major, having a monthly income between 500 and 1000 new Israeli shekel per month, whose studies were funded by their parents, and having mental problems. Our study would propose to conduct early screenings of university students who may be at risk for NES in order to develop appropriate interventions in an effort to decrease NES occurrence.

## Data Availability

Data and materials are available upon request and with permission of Dr. Manal Badrasawi at m.badrasawi@najah.edu.

## Abbreviations

NES: Night Eating Syndrome
DSM: Diagnostic and statistical manual of mental disorders
WHO: World Health Organization
IPAQ: International physical activity questionnaire
GHQ-12: 12-item general health questionnaire
NEQ: Night Eating Questionnaire
